# Novel order-level lineage of ammonia-oxidizing archaea widespread in marine and terrestrial environments

**DOI:** 10.1093/ismejo/wrad002

**Published:** 2024-01-10

**Authors:** Yue Zheng, Baozhan Wang, Ping Gao, Yiyan Yang, Bu Xu, Xiaoquan Su, Daliang Ning, Qing Tao, Qian Li, Feng Zhao, Dazhi Wang, Yao Zhang, Meng Li, Mari-K H Winkler, Anitra E Ingalls, Jizhong Zhou, Chuanlun Zhang, David A Stahl, Jiandong Jiang, Willm Martens-Habbena, Wei Qin

**Affiliations:** State Key Laboratory of Marine Environmental Science, College of the Environment and Ecology, Xiamen University, Xiamen 361005, China; Department of Microbiology, Key Laboratory of Agricultural and Environmental Microbiology, Ministry of Agriculture and Rural Affairs, College of Life Sciences, Nanjing Agricultural University, Nanjing 210095, China; Department of Microbiology, Key Laboratory of Agricultural and Environmental Microbiology, Ministry of Agriculture and Rural Affairs, College of Life Sciences, Nanjing Agricultural University, Nanjing 210095, China; National Library of Medicine, National Institutes of Health, Bethesda, MD 20894, United States; Department of Ocean Science and Engineering, Shenzhen Key Laboratory of Marine Archaea Geo-Omics, Southern University of Science and Technology, Shenzhen 518055, China; Shanghai Sheshan National Geophysical Observatory , Shanghai 201602, China; College of Computer Science and Technology, Qingdao University , Qingdao 266101, China; School of Biological Sciences, Institute for Environmental Genomics, University of Oklahoma, Norman, OK 73019, United States; School of Biological Sciences, Institute for Environmental Genomics, University of Oklahoma, Norman, OK 73019, United States; State Key Laboratory of Marine Environmental Science, College of Ocean and Earth Sciences, Xiamen University, Xiamen 361005, China; CAS Key Laboratory of Urban Pollutant Conversion, Institute of Urban Environment, Chinese Academy of Sciences, Xiamen 361021, China; State Key Laboratory of Marine Environmental Science, College of the Environment and Ecology, Xiamen University, Xiamen 361005, China; State Key Laboratory of Marine Environmental Science, College of Ocean and Earth Sciences, Xiamen University, Xiamen 361005, China; Archaeal Biology Center, Institute for Advanced Study, Shenzhen University, Shenzhen 518060, China; Department of Civil and Environmental Engineering, University of Washington, Seattle, WA 98195, United States; School of Oceanography, University of Washington, Seattle, WA 98195, United States; School of Biological Sciences, Institute for Environmental Genomics, University of Oklahoma, Norman, OK 73019, United States; School of Civil Engineering and Environmental Sciences, University of Oklahoma, Norman, OK 73019, United States; Department of Earth and Environmental Sciences, Lawrence Berkeley National Laboratory, Berkeley, CA 94720, United States; Department of Ocean Science and Engineering, Shenzhen Key Laboratory of Marine Archaea Geo-Omics, Southern University of Science and Technology, Shenzhen 518055, China; Shanghai Sheshan National Geophysical Observatory , Shanghai 201602, China; Department of Civil and Environmental Engineering, University of Washington, Seattle, WA 98195, United States; Department of Microbiology, Key Laboratory of Agricultural and Environmental Microbiology, Ministry of Agriculture and Rural Affairs, College of Life Sciences, Nanjing Agricultural University, Nanjing 210095, China; Department of Microbiology and Cell Science, Fort Lauderdale Research and Education Center, University of Florida, Davie, FL 33314, United States; School of Biological Sciences, Institute for Environmental Genomics, University of Oklahoma, Norman, OK 73019, United States

**Keywords:** *Nitrosomirales*, ammonia-oxidizing archaea, nitrification, subsurface, sponge, formate metabolism, nitrate reduction

## Abstract

Ammonia-oxidizing archaea (AOA) are among the most ubiquitous and abundant archaea on Earth, widely distributed in marine, terrestrial, and geothermal ecosystems. However, the genomic diversity, biogeography, and evolutionary process of AOA populations in subsurface environments are vastly understudied compared to those in marine and soil systems. Here, we report a novel AOA order *Candidatus* (*Ca.*) Nitrosomirales which forms a sister lineage to the thermophilic *Ca.* Nitrosocaldales. Metagenomic and 16S rRNA gene-read mapping demonstrates the abundant presence of *Nitrosomirales* AOA in various groundwater environments and their widespread distribution across a range of geothermal, terrestrial, and marine habitats. Terrestrial *Nitrosomirales* AOA show the genetic capacity of using formate as a source of reductant and using nitrate as an alternative electron acceptor. *Nitrosomirales* AOA appear to have acquired key metabolic genes and operons from other mesophilic populations via horizontal gene transfer, including genes encoding urease, nitrite reductase, and V-type ATPase. The additional metabolic versatility conferred by acquired functions may have facilitated their radiation into a variety of subsurface, marine, and soil environments. We also provide evidence that each of the four AOA orders spans both marine and terrestrial habitats, which suggests a more complex evolutionary history for major AOA lineages than previously proposed. Together, these findings establish a robust phylogenomic framework of AOA and provide new insights into the ecology and adaptation of this globally abundant functional guild.

## Introduction

The ammonia-oxidizing archaea (AOA) represent one of the most abundant and ubiquitous archaeal groups in the global biosphere [1-3]. They account for nearly 30% of microbial plankton in the oceans and up to 5% of microbial populations in soils [4, 5]. As such, the AOA represent a biogeochemically significant functional guild that plays an important role in the global nitrogen cycle [6]. They are almost exclusively responsible for ammonia oxidation in oligotrophic marine environments and contribute as much as 80% of the emission of ozone-depleting potent greenhouse gas nitrous oxide from the oceans [7-10]. This globally abundant and ecologically significant group of archaea was assigned to a major archaeal phylum *Thaumarchaeota* (also named as *Nitrososphaerota*) [11-13].

Although all AOA are united by a common physiology of chemoautotrophic growth on ammonia oxidation and carbon fixation, the extensive genetic repertoire of their pan-genome suggests that many additional adaptive features are associated with the remarkable ecological success of this functional guild as reflected by their high global abundance and wide niche breadth [14-20]. Previous AOA phylogenetic, ecological, and evolutionary analyses were based on a standardized taxonomic framework that divided ammonia-oxidizing *Thaumarchaeota* into four major lineages, the *Nitrosopumilales* (Group 1.1 a) [21], *Candidatus* (*Ca*.) Nitrosotaleales (Group 1.1 a-associated, now reclassified as a family within the *Nitrosopumilales*) [22], *Nitrososphaerales* (Group 1.1 b) [23], and *Ca.* Nitrosocaldales (thermophilic AOA, ThAOA) [24], that appear somewhat specialized, respectively, to aquatic (marine or freshwater), acidic soil, neutral or alkaline soil, and geothermal environments [15, 16, 19, 20, 25, 26].

A recent metagenomic study of cold deep seawater (~2.3°C) recovered from the Mariana Trench yielded a metagenome-assembled genome (MAG) that appeared to be phylogenetically closely associated with the deeply branching ThAOA [27]. This marine AOA MAG, along with two other closely related MAGs recovered from deep-sea waters (UBA213 and SAT137) [28, 29], had been assigned to *Ca*. Nitrosocaldales based on the previously established phylogenetic backbone of AOA [14, 15]. However, further phylogenomic analyses with additional genomes that represent a broader range of genotypes of this *Ca*. Nitrosocaldales-associated lineage are needed to resolve and substantiate the uncertain taxonomic affiliation and evolutionary history of basal AOA clades. In addition, how widespread these *Ca*. Nitrosocaldales-associated mesophilic AOA are in marine and terrestrial environments and their diversity, metabolic adaptation, and ecological significance remain unknown.

Here, we conduct phylogenomic and comparative genomic analyses of 161 AOA and non-ammonia-oxidizing *Thaumarchaeota* genomes, including 23 MAGs and single amplified genomes (SAGs) of this understudied group that were obtained from a variety of subsurface, geothermal, soil, and marine environments. We show that these 23 MAGs and SAGs form a highly supported monophyletic order-level lineage within the *Nitrososphaeria*, which we, here, designate as a new AOA order *Ca*. Nitrosomirales. The global distribution of this previously undefined AOA order was investigated by extensively searching for *Ca*. Nitrosomirales gene sequences in marine and groundwater metagenome datasets as well as in the Microbiome Search Engine 2 (MSE2) database that contains over 300 000 samples sequenced from a wide range of natural and manmade ecosystems [30]. Our findings provide new understanding of the metabolic potential and biogeography of the *Ca*. Nitrosomirales order that appears to represent an abundant AOA genotype in previously undersampled habitats, such as many terrestrial subsurface environments and deep-sea sponges.

## Materials and methods

### The identification and assembly of *Nitrosomirales* AOA genomes

This study encompassed a total of 23 *Nitrosomirales* AOA genomes, with 22 *Nitrosomirales* MAGs and SAGs obtained from public databases, and one MAG (WS3) assembled and binned as part of this investigation. The initial identification of the *Nitrosomirales* clade stemmed from a preliminary phylogenetic analysis, wherein three previously reported marine AOA MAGs SAT137, UBA213, and MTA3 were clustered as a lineage that was associated with thermophilic *Ca*. Nitrosocaldales AOA. Subsequently, BLASTn searches were performed using the criteria of sequence identity ≥90% and E-value ≤ 1 × e^−10^ to identify *Nitrosomirales*-like 16S rRNA and *amoA* (encoding the alpha subunit of ammonia monooxygenase, AMO) genes in the JGI metagenome and scaffold databases as well as the NCBI Refseq database. The phylogenetic affiliation of these additional *Nitrosomirales* MAGs and SAGs was further confirmed through phylogenomic analysis. Only those that clustered with SAT137, UBA213, and MTA5 as a monophyletic group were designated as *Nitrosomirales* AOA and selected for downstream comparative analysis.

*Nitrosomirales* MAG WS3 was retrieved from a metagenome dataset of a warm spring in Beatty, Nevada (JGI project ID: 3300025157) and was assembled in this study. The Illumina sequence reads downloaded from the JGI database were trimmed and filtered using Trimmomatic (version 0.36) [31] to remove low-quality reads. The quality-filtered reads were assembled using MEGAHIT (version 1.2.9) [32] with a range of k-mers (k = 21, 33, 55, 77, 99, 127). Contigs longer than 500 bp were then binned using MetaBAT (version 1.7) [33]. *Nitrosomirales* MAG WS3 was identified by mapping *amoA* genes to an in-house AOA *amoA* gene database (Supplementary Dataset S1) that includes the AOA *amoA* gene OTUs (operational taxonomic units) previously identified by Alves *et al*. [25], as well as the *amoA* genes retrieved from the other 22 *Nitrosomirales* MAGs reported in this study. Its affiliation was further confirmed through phylogenomic tree construction (see more details below in the phylogenomic tree construction methods). The genes in the genomes of both *Ca*. Nitrosomirales sp. WS3 and *Ca*. Nitrosomirales sp. UBA213, the latter of which has not been annotated in public databases, were predicted using GeneMarkS (version 4.30) [34] and subsequently annotated by the NCBI Prokaryotic Genome Annotation Pipeline [35] (Supplementary Datasets S2 and S3).

### The distribution of *Candidatus* Nitrosomirales AOA at global scale

The 16S rRNA gene sequences of all AOA genomes were extracted by the ssu_finder of CheckM (version 1.0.12) [36]. We searched the extracted AOA 16S rRNA gene sequences against the MSE2 database as previously described [30]. The MSE2 database contains more than 300 000 16S rRNA gene amplicon and metagenomic sequencing samples collected from marine and terrestrial habitats as well as human, animal, and plant-associated microbiomes. AOA-containing microbiomes in MSE2 were identified via VSEARCH (version 2.22.1) using a 97% sequence similarity cut-off, by comparing the amplicon and metagenomic sequences to the 16S rRNA gene sequences extracted from 134 AOA genomes (Supplementary Table S1) [37]. The relative abundance of AOA amplicon sequences in each sample was normalized using the Meta-Storms algorithm [38] to reduce the 16S rRNA gene amplification bias.

To explore the distribution of *Nitrosomirales* AOA in the global ocean, we searched *amoA* gene reads from global ocean metagenomic databases, including *Tara*-Oceans (2009–13) [39] and *Malaspina*-2010 metagenomic data [40], Hawaii Ocean time series station metagenomic data [41], and the Mariana Trench metagenomic data [42]. To confirm the prevalence of *Nitrosomirales* AOA in groundwaters, we conducted competitive fragment recruitment to determine the relative recruitment to *Nitrosomirales* genomes in the groundwater metagenomes. Specifically, we examined metagenomic samples collected from the Rifle research site (FP-101), located adjacent to the Colorado River [43], as well as contaminated groundwater samples collected from the Oak Ridge Integrated Field Research Challenge (OR-IFRC) experimental sites [44]. Briefly, we first trimmed the raw sequencing reads of metagenomic samples by Trimmomatic (version 0.36) [31]. The trimmed reads were then mapped via Diamond (version 0.9.24.125) [45] with a threshold of 80% sequence similarity and 100 bp coverage, as well as an E-value cut-off of 1 × e^−10^, to an in-house AOA species genome database (Supplementary Table S1). The recruited reads were further assigned to *Nitrosomirales* and other major AOA lineages, and then normalized by the average genomic sizes (Mb) of each lineage. In addition, we searched for 16S rRNA and *amoA* gene sequences of *Nitrosomirales* in the NCBI database with BLASTn using a sequence similarity cut-off of 95% and 90%, respectively, and an E-value cut-off of 1 × e^−10^. The phylogenetic affiliation of the identified sequences was further confirmed in the 16S rRNA and *amoA* gene trees. Only the sequences affiliated with a bootstrap value of over 85% (out of 1000 replications) within the *Nitrosomirales* clade were identified as the members of *Nitrosomirales*.

### Phylogenomic tree construction

**Figure 1:**
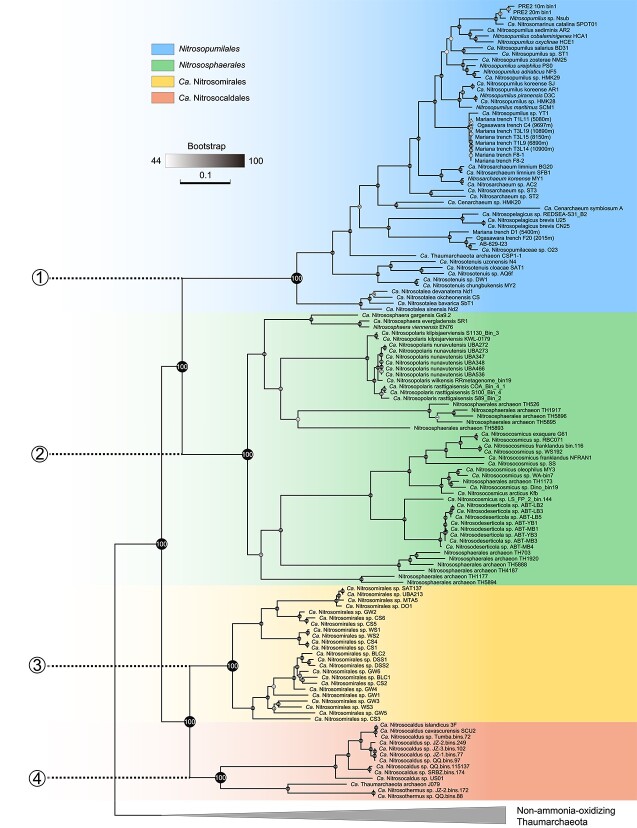
Phylogeny of *Ca*. Nitrosomirales and other AOA orders; phylogenomic inference of AOA species affiliated to the orders *Nitrosopumilales* (blue, basal lineage #1), *Nitrososphaerales* (green, basal lineage #2), *Ca*. Nitrosomirales (yellow, basal lineage #3), and *Ca*. Nitrosocaldales (orange, basal lineage #4) based on concatenated sequences of 70 conserved single-copy core genes; the non-ammonia-oxidizing thaumarchaeotal genomes were used as outgroups (gray); please note that, based on the recent comparative phylogenetic analysis of AOA genomes [15], the previously defined acidophilic AOA group *Ca.* Nitrosotaleales has been merged with *Nitrosopumilales*; phylogenomic tree was constructed using the protein mixture model UL3 (three-matrix model); confidence values were provided based on 1000 bootstrap replications.

Phylogenomic tree construction was based on concatenated alignments of 70 conserved marker genes [46] (Supplementary Table S2) from 134 AOA genomes, along with an outgroup of non-ammonia-oxidizing *Thaumarchaeota* genomes [47] (Supplementary Table S1). The 70 marker genes were identified by BLASTp, and they were individually aligned by MAFFT (version 7.221) [48]. Subsequently, the conserved regions of alignments were extracted by Gblocks (version 0.91b) [49]. Conserved regions were concatenated as a single evolutionary unit for phylogenomic tree construction. The maximum likelihood AOA phylogenomic tree was built with concatenated sequences via IQ-TREE (version 2.1.2 COVID-edition) [50], and branch support was assessed using 1000 ultrafast bootstrap replicates. The constructed phylogenomic trees, using both the protein mixture model UL3 (three-matrix model) [51] (Fig. 1) and the best-fit model (LG + F + R9, single amino acid replacement rate matrix model) (Supplementary Fig. S1), showed consistent order-level tree topology.

### Comparative genomic analysis and pathway construction

Average nucleotide identity (ANI) was calculated by pyani (version 0.2.12) [52] using BLASTn alignment. The percentage identity and alignment coverage between each of two genomes were displayed in heatmap format. Average amino acid identity (AAI) between two AOA genomes was calculated by EzAAI (version 1.2.1) [53]. AOA proteins were clustered into orthogroups by OrthoFinder (version 2.5.4) [54]. The thresholds of orthogroups were set to achieve a pairwise coverage of 50% and sequence identity of 50% based on all-against-all BLASTp. The AOA genomes with over 50% completeness were used for core and pan-genome analysis. Core genome represents the orthogroup genes shared by all species genomes, MAGs, and SAGs, and pan-genome represents the collective set of genes present in at least one genome. For *m* selected out of n genomes, a total of *n*!/[*m*!·(*n–m*)!] combinations were calculated to determine the sizes of the core and pan-genomes. Up to 5000 random combinations were sampled for core genome and pan-genome analyses. To compare the pan-genome openness of different AOA orders, the average number of new unique genes per Mbp genome was calculated with the sequential addition of each AOA genome, and the core and pan-genomes were fitted using Heaps’ law. Finally, the pan-genomes of *Nitrosomirales* and other AOA orders were visualized using the Anvi’o software (version 7.1) [55]. The orthogroups enriched in *Nitrosomirales* AOA were manually labeled based on their clustering distance and annotated using the archaeal clusters of orthologous genes (arCOGs) database [56]. The representative sequence for each orthogroup was randomly selected and then mapped to the reference sequence in the arCOGs database using BLASTp with a threshold of 50% sequence identity and an E-value cut-off of 1 × e^−5^.

The functions of all orthogroups in *Nitrosomirales* were annotated using the Nr, KEGG, and arCOG databases, and organized and compiled based on previous comparative genomics studies of marine [14, 57-59], soil [60], and hot spring [61, 62] AOA species. The putative metabolic pathways were classified into 13 groups, including ammonia oxidation/assimilation/nitrate reduction, urea utilization, carbon fixation/metabolism, sulfur assimilation/metabolism, phosphorus utilization, stress response, thermo(osmo)-adaptation, amino acids/vitamins/cofactors, information processes, S-layer synthesis, lipid biosynthesis, glycosyl transferase, and transporters. Putative transporters were further identified by screening against the Transporter Classification Database [63]. The annotations of enzymes that are potentially involved in the formation and break down of carbohydrates were further confirmed by searching against the Carbohydrate-Active enZYme Database (CAZy) [64].

### Phylogenetic analysis of key functional genes

The sequences of 16S rRNA, *amoA*, *atpA*, and *atpC* (encoding the alpha and epsilon subunits of ATPase, respectively), *nirK* (encoding putative copper-dependent nitrite reductase), and *ureC* genes (encoding the alpha subunit of urease) were extracted from the collected 134 AOA genomes (Supplementary Table S1). Additional *Nitrosomirales* 16S rRNA genes and 33,387 AOA *amoA* genes were collected from NCBI and extracted from a previous *amoA* gene-based phylogenetic analysis [25], respectively, for comparative phylogenetic analysis. It is worth noting that when aligning the *Nitrosomirales* AOA *amoA* genes with one of the most widely used *amoA* gene primers (Arch-amoAF and Arch-amoAR) [1], we identified five mismatches in the reverse primer alignment region. This points out the need for designing new primers when using amplification-based methods to study the distribution of *Nitrosomirales* AOA in different environments.

The phylogenetic trees of the 16S rRNA and other key functional genes of AOA were built using IQ-TREE (version 2.1.2 COVID-edition) with the best-fit model selection [50]. Briefly, gene sequence alignment was carried out using MAFFT (version 7.407) [48] and then edited with Gblocks (version 0.91b) [49] to identify conserved regions. The best-fit model of evolution for each gene set was selected by ModelFinder [65] with “-m MF –T AUTO” flag as follows: GTR + F + R4 for 16S rRNA genes, GTR + F + I + Γ4 for *amoA* genes, LG + R5 for *atpA* genes, LG + R4 for *atpC* genes, WAG+F + G4 for *nirK* genes, and LG + R3 for *ureC* genes. Subsequently, the maximum likelihood phylogenetic trees were built using IQ-TREE (version 2.1.2 COVID-edition) with the best-fit models mentioned above and 1000 times ultrafast bootstrap replicates [66]. The non-ammonia-oxidizing *Thaumarchaeota* were used as the outgroup for the 16S rRNA gene tree. *Thermoproteales*, *Sulfolobus*, *Desulfurococcales Ignicoccus hospitalis* KIN4/I, *Ca*. Bathyarchaeota archaeon ex4484_231, and *Methanosuratus*/*Methanomethylicus* were used as the outgroups for the phylogenetic trees of the A and C subunits of the A-type ATPases, and *Thermoplasmatales* as well as *Enterococcus hirae* ATCC 9790 were used as the outgroups for the trees of the A and C subunits of the V-type ATPases.

The FdhA (alpha subunit of NAD^+^-dependent formate dehydrogenase) and NarG (alpha subunit of nitrate reductase) protein clusters of *Nitrosomirales* AOA, consisting of 6 sequences and 8 sequences, respectively, were aligned with the reference sequences from UniProt database using MAFFT (version 7.407) [48]. Maximum likelihood phylogenetic trees were constructed for FdhA and NarG using the best-fit models LG + R5 and LG + R6, respectively.

### Availability of data and materials

AOA genomes used in this study are available in the NCBI and JGI databases. The corresponding accession numbers are listed in Supplementary Table S1. The GFF files of *Nitrosomirales* MAGs UBA213 and WS3 are provided in Supplementary Datasets S2 and S3, respectively. The contig sequences of MAG UBA213 can be obtained from the NCBI database (GCA_002494485.1), and the contig sequences of MAG WS3 can be found in Supplementary Dataset S4. Completeness, contamination, and coding density of AOA genomes were assessed by CheckM (version 1.0.12) [36]. All other data products associated with this study are available from the corresponding authors upon request.

## Results and discussion

### *Nitrosomirales* represents an order-level lineage of AOA

We compiled a 161-genome dataset that comprises 134 cultured AOA species genomes, MAGs, and SAGs as well as 27 representative genomes of non-ammonia-oxidizing *Thaumarchaeota* to serve as outgroups (Supplementary Table S1). To infer the most probable evolutionary relationship among AOA taxa and resolve the uncertain taxonomic affiliation of mesophilic members that were affiliated to *Ca*. Nitrosocaldales, we constructed a maximum likelihood phylogenomic tree from the concatenation of 70 conserved marker genes (Supplementary Table S2) present in AOA genomes and outgroup thaumarchaeotal genomes. We obtained 20 additional MAGs and SAGs from various terrestrial subsurface, deep soil, and marine habitats that were clustered with three previously reported marine AOA MAGs MTA5, UBA213, and SAT137 [27-29], and these 23 genomes together formed a well-supported monophyletic group branching as a sister clade to the thermophilic *Ca.* Nitrosocaldales AOA (Fig. 1).

Among these newly obtained MAGs and SAGs, nine were retrieved from warm/thermal spring (WS1–WS3) and carbonate spring (CS1–CS6) waters and sediments, six originated from groundwaters (GW1–GW6), two from basaltic lava caves (BLC1 and BLC2), two from deep sandy soils in switchgrass fields (DSS1 and DSS2), and one from deep ocean waters (DO1) (Supplementary Table S3). Eleven of these genomes showed a generally high level of completeness (>78%) and low contamination (<5%) (Supplementary Table S3). MAGs DSS1 and WS1 are complete or nearly complete genomes and estimated to be 100.0% and 99.9% complete with minimal contamination (1.0%), respectively. Along with the three previously reported marine AOA MAGs [27-29], these MAGs and SAGs span a wide size range from 1.0 to 3.7 Mbp. Similar to other AOA genomes, among these 23 genomes, the GC content of those with marine origins (34.6%–36.1%) was lower than that of those originating from terrestrial environments (40.3%–47.7%) (Supplementary Table S3). The ANI and the AAI values among these 23 genomes were 67.6%–99.6% (Supplementary Table S4) and 68.2%–99.9% (Supplementary Table S5), respectively, reflecting generally high genomic diversity within this group. The characterized *Nitrosomirales* genomes shared low genomic homology (<65.7% ANI and <61.4% AAI) and alignment fraction (4.5%–26.6%) with other marine and terrestrial AOA genomes (Supplementary Tables S4 and S5).

Additional whole-genome based taxonomic analysis using the Genome Taxonomy Database Toolkit also showed these 23 MAGs and SAGs to be members of a distinct lineage along with the other three formally described major AOA lineages (Supplementary Table S3) [21, 23, 24]. We here propose the name *Ca.* Nitrosomirales to represent this AOA order within the class *Nitrososphaeria* (Fig. 1), which forms a sister lineage to thermophilic *Ca*. Nitrosocaldales, and the complete MAG DSS1 from deep sandy soil to represent the candidate family *Nitrosomiraceae* and genus *Nitrosomirus*. The name *Nitrosomirus* refers to the organisms as ammonia oxidizers with the capacity of oxidizing ammonia to nitrite (from the Latin “nitrosus,” full of natron; here intended to mean nitrous) and its wide distribution range in various marine, soil, geothermal, and subsurface habitats (from the Latin adjective “mirus,” meaning surprising and amazing), spanning a range of temperature, salinity, pressure, and nutrient availability (see the description below).

### *Nitrosomirales* AOA are widely distributed in diverse terrestrial, marine, and geothermal habitats

**Figure 2:**
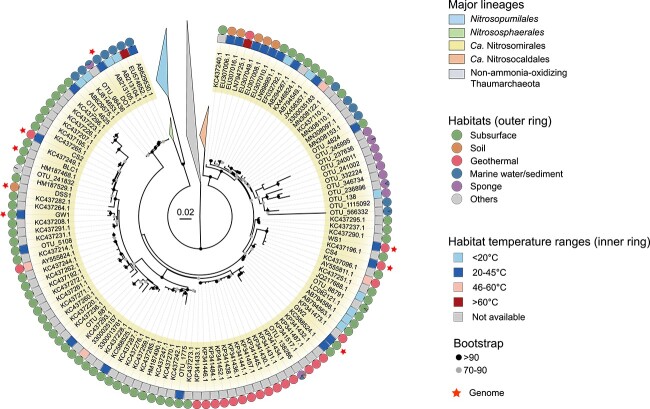
Phylogenic distribution of *Ca*. Nitrosomirales 16S rRNA genes according to habitat types (circles, outer ring) and temperature ranges (squares, inner ring); the scale bar represents 2% estimated sequence divergence; the 16S rRNA sequences that retrieved from *Ca*. Nitrosomirales genomes were indicated with stars; the 16S rRNA genes of other thaumarchaeotal lineages were collapsed as triangles.

**Figure 3:**
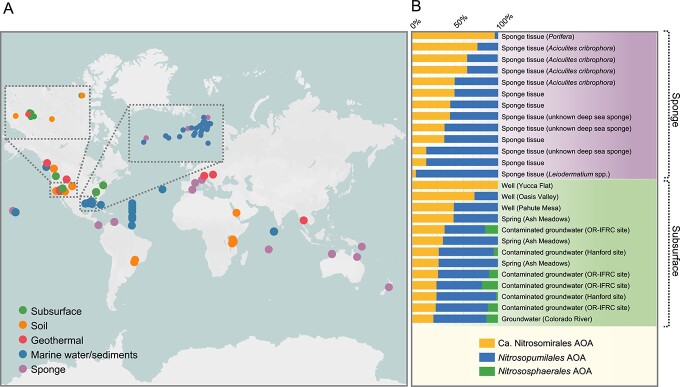
The global distribution of *Nitrosomirales* AOA in various terrestrial and marine environments; (A) *Nitrosomirales* AOA were found to be abundant in some sponge-associated microbiomes and subsurface environments; (B); the relative abundance of *Nitrosomirales* AOA was estimated by comparing the number of amplicon sequencing and metagenomic reads that mapped to the 16S rRNA and *amoA* genes as well as the whole genomes of *Nitrosomirales* AOA relative to those of total AOA in the MSE2 global 16S rRNA gene database, global ocean metagenome databases, and groundwater metagenomes.

The recovery of many *Nitrosomirales* MAGs from subsurface, deep soil, deep ocean, and geothermal environments suggests that *Nitrosomirales* AOA are widely distributed across a range of terrestrial and marine habitats. To further explore the distribution of this understudied group in the global biosphere, we searched the *Nitrosomirales* MAG-derived 16S rRNA sequences against the MSE2 database that contains over 300 000 16S rRNA gene amplicon and metagenomic sequencing samples obtained from a broader range of natural habitats and engineered systems, as well as human, animal, and plant-associated microbiomes (see Materials and Methods for details) [30]. *Nitrosomirales*-derived 16S rRNA gene sequences were found in various terrestrial and marine habitats, including groundwaters, grassland soils, agricultural soils, stromatolite mats, marine sponges, and marine sediments (Fig. 2 and Supplementary Table S6). Additional extensive searches of 16S rRNA (sequence identity cut-off of 95% and E-value cut-off of 1 × e^−10^) in the NCBI database further identified *Nitrosomirales* sequences in (moderately) thermophilic habitats, such as hydrothermal vents (72–103°C), hot springs (55°C), and thermal karst well waters (73.7°C) (Fig. 2 and Supplementary Table S6). Both the 16S rRNA and *amoA* gene-based phylogenies support the monophyletic grouping of *Nitrosomirales* metagenomic, amplicon, and clone sequences (Fig. 2 and Supplementary Fig. S2). Together, our results indicate that the habitats of *Nitrosomirales* AOA include a wide variety of terrestrial, marine, subsurface, and geothermal environments (Fig. 3A).

### *Nitrosomirales* AOA represent an abundant AOA lineage in subsurface habitats and deep-sea sponges

Although the first three *Nitrosomirales* MAGs were recovered from the dark ocean, *Nitrosomirales* AOA were rarely detected in global ocean metagenomes. We assessed the number of metagenomic reads mapping to the *amoA* genes of *Nitrosomirales* AOA and other marine AOA genotypes in global ocean metagenome databases across four oceans and two seas, spanning from epipelagic to hadopelagic zones [39-42], and found that *Nitrosomirales* AOA only comprised at most 0.5% of the total AOA community in well-studied ocean waters (Supplementary Table S7). In contrast, the 16S rRNA gene and whole genome read recruitment showed that *Nitrosomirales* AOA can represent an abundant AOA genotype or even exclusively represent the whole AOA community in some previously undersampled habitats, such as deep-sea sponges and groundwaters (Fig. 3B and Supplementary Tables S6 and S7).

Marine AOA have been frequently reported to associate with sponges [67-71] and often dominate the whole archaeal communities of sponge microbiomes [72]. Since the sequencing of the first sponge AOA symbiont, *Ca.* Cenarchaeum symbiosum, hosted by the demosponge *Axinella mexicana* [67], additional AOA MAGs have been obtained from various shallow-water and deep-sea sponge-associated microbiomes. Marine sponges are a highly diverse clade of metazoans that contains 125 families, 680 genera, and 11 000 species [73]. The molecular surveys of sponge microbiota indicated that microbial communities were mostly specific to sponge species [74], and the dominant AOA populations were even specific to individual sponges [71]. Previous studies of thaumarchaeotal sponge symbionts only focused on several limited sponge families of the classes *Demospongiae* and *Hexactinellida*. These sponge symbionts were affiliated with five different AOA genera, *Nitrosopumilus* [69], *Ca*. Cenarchaeum [67], *Ca.* Nitrosopelagicus [71], *Ca.* Nitrosospongia [68], and *Ca.* Cenporiarchaeum [69], all of which were within the order *Nitrosopumilales*.

We found that, in addition to *Nitrosopumilales*, the members belonging to *Nitrosomirales* can also constitute a significant fraction (up to 96.1%) of the total AOA populations in deep-sea sponges (Fig. 3B). *Nitrosomirales* AOA were specifically hosted by the deep-sea (~200–550 m) *Aciculites* sponge species within the *Scleritodermidae* family and the *Porifera* phylum (Supplementary Table S6). This lineage of sponges typically inhabits circumtropical and subtropical regions [75], and it was poorly represented in the previous 16S rRNA gene surveys and the global sponge microbiome metagenome database. In addition, the 16S rRNA gene sequences that are closely related to *Ca*. Nitrosocosmicus AOA within the *Nitrososphaerales* were found in the microbiomes of marine sponges *Spirastrella panis* [76], *Astrosclera willeyana* [77], *Theonella swinhoei* [78], *Pseudoceratina purpurea* (NCBI accession No.: KU064739), and *Halichondria oshoro* (HM101091) (Supplementary Fig. S3). These findings significantly expand the genetic diversity of the sponge-associated marine AOA beyond the order *Nitrosopumilales*. It is very likely that *Nitrosomirales* AOA play an important role in the nitrogen metabolism and nitrogenous waste removal of deep-sea *Aciculites* sponges, similar to other characterized sponge-associated *Nitrosopumilales* AOA [67-69].

Another relatively undersampled AOA habitat is the terrestrial subsurface environment. The biogeography of AOA in groundwater and cave ecosystems, as well as the associated environmental variables that control the abundance and composition of AOA communities in these systems, is poorly documented [79]. Given that several *Nitrosomirales* MAGs were retrieved from the Death Valley Regional Flow System (DVRFS), we leveraged the available 16S rRNA amplicon sequencing data [80] collected from the DVRFS region to assess the relative abundance of *Nitrosomirales* AOA across three major groundwater basins (Supplementary Table S6). *Nitrosomirales* AOA 16S rRNA genes were detected in nine DVRFS groundwater sites out of the 36 total sampling sites. Based on 16S rRNA gene read recruitment, we found that *Nitrosomirales* AOA can constitute a substantial proportion (30.9%–100.0%) of the total AOA populations in six of these groundwater sites (Fig. 3B and Supplementary Table S6). *Nitrosomirales* AOA were detected in both shallow (0–25 m sampling depth) and deep (474–700 m sampling depth) aquifers with distinct aquatic geochemistry, including Ca-Mg-HCO_3_, Na-HCO_3_, and NaCl-dominated groundwaters [80] (Supplementary Table S6). In addition, we used competitive fragment recruitment to estimate the relative recruitment to *Nitrosomirales* AOA genomes in the metagenome datasets obtained from an aquifer adjacent to the Colorado River [43]. *Nitrosomirales* AOA were detected in all eight groundwater samples, and they accounted for 24.8% of the total AOA populations in the sample GW2011-A-0.1 (Supplementary Table S7). *Nitrosomirales* AOA were also found in basaltic lava caves (Supplementary Table S3), together indicating their wide distribution in terrestrial subsurface environments.

We also identified this lineage in the 16S rRNA gene and metagenome datasets collected from the groundwaters at the Hanford site and the OR-IFRC site, respectively (Fig. 3B and Supplementary Tables S6 and S7), legacies of the Manhattan Project contaminated with mixed waste, including metals, radionuclides, and nitrate [81]. *Nitrosomirales* AOA can be found in uranium-contaminated groundwaters with high nitrate concentrations (as high as 9068.7 mg/l nitrate) (Supplementary Table S7). It is conceivable that respiratory ammonifiers with the capacity of the dissimilatory nitrate reduction to ammonium could supply ammonia to *Nitrosomirales* AOA and other archaeal and bacterial nitrifiers in these contaminated groundwater sites [82], and thus together these microbial communities may contribute to nitrogen transformation in terrestrial subsurface ecosystems.

### Genomic features and metabolic potential of *Nitrosomirales* AOA

**Figure 4:**
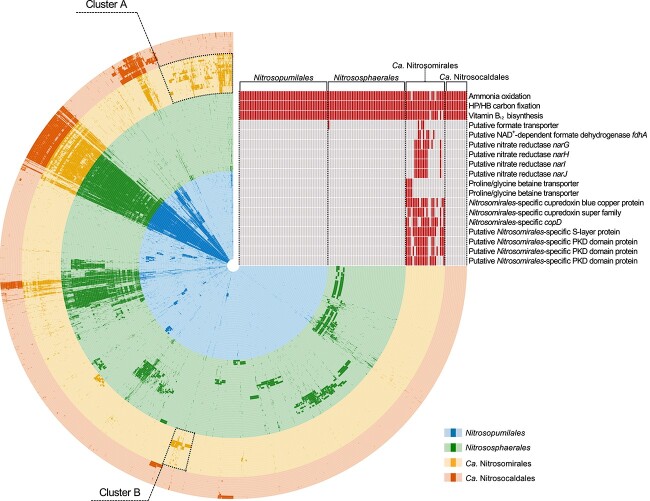
Anvi’o representation of the pangenome of *Nitrosomirales* and other AOA genomes; each radial layer represents an AOA genome, which was arranged by the order of phylogenomic tree shown in Fig. 1; in each layer, dark and light colors represent the presence and absence of protein clusters, respectively; the enriched protein clusters A and B in *Nitrosomirales* were indicated by the dashed frames on the genome map rails (see Supplementary Table S9 for the complete list of arCOGs annotations of these two enriched clusters); the heatmap in the top right corner represents the conserved functions present in the core genome of all AOA orders and highlights the key unique gene contents retrieved from the *Nitrosomirales*-enriched protein cluster A; the red color indicates the presence of the genes, while the gray color indicates their absence.

We calculated the core genome and pan-genome of *Nitrosomirales* and other AOA orders to get quantitative insights into the conserved and flexible gene pools of ammonia-oxidizing *Thaumarchaeota* (Supplementary Fig. S4). Comparative genomic analysis showed that all terrestrial and marine *Nitrosomirales* AOA MAGs and SAGs harbored the conserved pathway genes that are involved in the characterized central metabolism of AOA, including the copper-dependent respiration and electron transfer systems, 3-hydroxypropionate/4-hydroxybutyrate autotrophic carbon fixation cycle, and the biosynthesis of the B vitamin cofactors thiamin (B_1_), riboflavin (B_2_), pantothenate (B_5_), pyridoxine (B_6_), biotin (B_7_), and cobalamin (B_12_) (Fig. 4 and Supplementary Table S8).

The pan-genome openness of *Nitrososphaerales* was the highest among the four AOA orders (Supplementary Fig. S4B), and the high genomic diversity was driven by the extensive lateral gene transfer and gene duplication events during *Nitrososphaerales* evolution [15]. The *Nitrosomirales* AOA pan-genome within the available dataset contains a total of 5409 genes, and the pan-genome graph shows that the sampling of their pan-genome has not yet reached saturation (Supplementary Fig. S4B). Upon normalizing to genome size, it is estimated that an average of 41 novel unique genes per Mbp genome can be identified with each new *Nitrosomirales* species sequenced, which is comparable to the number of new unique genes estimated for *Nitrosopumilales* AOA (Supplementary Fig. S4C). We further performed Anvi’o pan-genome analysis to assess and visualize the *Nitrosomirales*-specific gene content (Fig. 4). A total of 298 314 coding sequences of the collected AOA genomes were clustered into 17 691 orthologous groups (OGs). Of these, 1637 OGs were specifically enriched in *Nitrosomirales* genomes but absent or scarce in other AOA orders (Fig. 4), including the accessory and unique genes that were assigned to certain functional categories of the arCOGs database, such as coenzyme transport and metabolism, energy production and conversion, cell wall/membrane/envelope biosynthesis, and amino acid transport and metabolism, as well as many hypothetical genes with unknown functions (Supplementary Table S9).

Different from the members of their sister order *Ca*. Nitrosocaldales [61, 62, 83], *Nitrosomirales* AOA lacked identifiable hydrogenase genes involved in potential hydrogen oxidation for energy conservation (Supplementary Table S8). Similar to *Nitrosocaldales* AOA, *Nitrosomirales* AOA also lack the genes encoding both the small and large subunits of the D-family DNA polymerase (PolD) (Supplementary Fig. S5), which are considered core genes in all other mesophilic AOA lineages [61, 62, 83]. Instead, only genes for B-family (PolB; OG00206) and Y-family (PolY; OG00161) DNA polymerases were identified in *Nitrosomirales* AOA (Supplementary Fig. S5 and Supplementary Table S8). Given that the *in vitro* activities of PolB and PolY DNA polymerases haven been confirmed in the PolD-lacking crenarchaeon *Sulfolobus acidocaldarius* [84], it is conceivable that *Nitrosomirales* and *Nitrosocaldales* AOA may share a similar DNA replication machinery centered around PolB and PolY.

Among the *Nitrosomirales*-enriched genes, the genes that encode the alpha (major, containing the active site) subunit of a soluble NAD^+^-dependent formate dehydrogenase (*fdhA*, OG05616) were found in some *Nitrosomirales* genomes, including those obtained from carbonate and warm springs and deep sandy soils (Fig. 4 and Supplementary Table S8). The FdhA proteins of *Nitrosomirales* AOA were found to be phylogenetically most closely related to those of *Sulfolobaceae* (Supplementary Fig. S6A). *Nitrosomirales* genomes also encode the beta (OG02267) and gamma (OG02824) subunits of the putative NAD^+^-dependent Fdh, which may be involved in electron transfer processes and regulatory functions (Supplementary Table S8). Likewise, the beta subunit of the putative NAD^+^-dependent Fdh was also identified in the NP-theta and NP-iota clades of *Nitrosopumilales* AOA recovered from deep-sea sediments [85]. However, unlike marine sedimentary *Nitrosopumilales* AOA, the terrestrial *Nitrosomirales* genomes contain putative formate transporter genes (OG10131) (Fig. 4 and Supplementary Table S8). Taken together, these results strongly suggest that terrestrial *Nitrosomirales* AOA may be able to uptake and utilize formate as an alternative electron donor and/or source of reductant.

It has been shown that *Nitrospira* nitrite-oxidizing bacteria (NOB) species were able to grow using formate as sole substrate with O_2_ or nitrate as terminal electron acceptors [86]. We identified genes that encode the alpha and beta subunits of the putative nitrate reductase (NarG and NarH, OG04231 and OG04232) in 11 terrestrial *Nitrosomirales* genomes (Fig. 4 and Supplementary Table S8). The *narG* sequences of *Nitrosomirales* AOA clustered with multiple sequences from *Natronococcus*, *Thermogutta*, *Thioploca*, *Thioalkalivibrio*, and *Hydrogenobacter* (Supplementary Fig. S6B). The putative *narI* (OG04233) and *narJ* (OG04234) genes were also found in proximity to the *narGH* genes, all of which are located within the same operon (Supplementary Fig. S7). Thus, it is tempting to speculate that the formate oxidation with nitrate reduction may be directly coupled in *Nitrosomirales* under anoxic conditions, as observed in NOB. The fact that *Nitrosomirales* AOA can be found in contaminated groundwater with high nitrate concentrations (Fig. 3B and Supplementary Table S7) implies that members of this lineage encoding nitrate reductase might derive benefits from utilizing the nitrate leached into the groundwaters. Future cultivation and isolation of *Nitrosomirales* AOA are required to confirm their metabolic capacity of using formate as a source of reductant and using nitrate as an alternative electron acceptor, and such metabolic versatility may enable *Nitrosomirales* AOA to survive during periods of ammonia and oxygen deprivation.

Genes encoding a putative endo α-1,4 polygalactosaminidase (OG13695) were identified in *Nitrosomirales* MAGs WS1 and WS2 (Supplementary Table S8). These homologs shared a 69.3% sequence identity and 85.1% coverage with the endo α-1,4 polygalactosaminidase from *Armatimonadota*, *Planctomycetota*, and *Deinococcus* strains. Additional examination using the CAZy database also classified these homologs of *Nitrosomirales* AOA as members of the endo α-1,4 polygalactosaminidase (the GH114 family of glucoside hydrolase), which can hydrolyze 1,4-polygalactosamine into oligosaccharides through an endo-acting mechanism [87]. However, no uptake system for polygalactosamine or polysaccharide was identified in *Nitrosomirales* AOA genomes. Only two putative ABC-type permeases for polysaccharide/polyol phosphate export system (OG04061 and OG06086) were encoded in *Nitrosomirales* AOA (Supplementary Table S8), and homologs of these putative exporters were also found in several *Nitrosopumilales* AOA species (*Nitrosopumilus piranensis* D3C, *Ca*. Nitrosopumilus sp. HMK28, and *Ca*. Nitrosotalea sinensis Nd2). Similar functional genes present in *Nitrososphaerales* AOA, implicated in *N*-acetylglucosamine hydrolysis and polysaccharide export, were suggested to be involved in cell surface modification and the production of extracellular polymeric substances in AOA [60].

The mesophilic *Nitrosomirales* AOA also contained several genes that were found in thermophilic *Ca*. Nitrosocaldus/Nitrosothermus AOA (Supplementary Table S8) and were associated with compatible solutes for thermoprotection and osmoprotection in (hyper)thermophiles, including the genes encoding mannosyl-3-phosphoglycerate synthase (OG01105) and cyclic 2, 3-diphosphoglycerate synthetase (OG02404) [61, 83, 88, 89]. Since homologous genes of mannosyl-3-phosphoglycerate synthase have been identified in other mesophilic and moderately ThAOA lineages as well [60, 61, 90], mannosyl-3-phosphoglycerate may primarily function as a compatible solute for osmoprotection rather than playing a significant role in thermoadaptation among AOA. In addition, all four obtained marine *Nitrosomirales* AOA MAGs encode putative ABC-type proline/glycine betaine transport systems (OG07687 and OG07688) (Supplementary Table S8). The annotations were further verified using the Transporter Classification Database [63]. This suggests the potential ability of marine *Nitrosomirales* AOA to uptake these common compatible solutes, aiding in the regulation of their osmotic balance in marine environments and providing resistance against various types of stress [91].

Other genes that are conserved in *Nitrosomirales* AOA include two unique small blue copper proteins that may function as electron shuttles in the respiratory system, one *Nitrosomirales*-specific CopD protein that may be involved in the regulation of copper homeostasis, and various regulatory proteins that may be associated with stress response and adaptation, such as *Nitrosomirales*-specific transcriptional regulatory and two-component regulatory proteins, and DNA repair proteins (Fig. 4 and Supplementary Tables S8 and S9). Putative surface layer (S-layer) protein genes were identified in *Nitrosomirales* genomes (Fig. 4 and Supplementary Table S8). Moreover, *Nitrosomirales* AOA were found to possess up to three additional putative PKD (polycystic kidney disease) domain protein homologs (Fig. 4 and Supplementary Table S8), which have been found in extracellular parts of archaeal S-layer proteins that assist in cell adhesion and intercellular interaction [92, 93].

### *Nitrosomirales* AOA acquire key metabolic genes via horizontal gene transfer

Similar to other AOA orders, *Nitrosomirales* AOA genomes contain genes encoding the known ABC subunits of AMO, the predicted AMOX, and newly identified AMOY and AMOZ subunits [94] (Fig. 4 and Supplementary Table S8). The electrons from ammonia oxidation are transferred to oxygen via the copper-dependent electron transfer chain, which would lead to the generation of a proton motive force for ATP synthesis via energy-yielding ATPase. The phylogenetic trees of AOA ATPase alpha (A) and epsilon (C) subunits showed clear bifurcating topologies of archaeal-type (A-type) and vacuolar-like (V-type-like) ATPase subgroups (Supplementary Fig. S8). The A-type ATPase gene clusters were found in both marine and terrestrial *Nitrosomirales* AOA genomes, and their phylogeny tracked organismal phylogeny rather than ecological habitat (Supplementary Fig. S8). Likewise, the A-type *atp* operon of marine *Nitrosomirales* AOA shared the conserved organization and orientation with those of the terrestrial *Ca*. Nitrosocaldales and *Nitrososphaerales* AOA, but were distinct from marine *Nitrosopumilales* species (Supplementary Fig. S9). Four terrestrial *Nitrosomirales* MAGs encoded the entire gene clusters for both A-type and V-type-like ATPases (Supplementary Fig. S9). Partial gene clusters of V-type ATPase were also found in the deep-sea MAG MTA5, two additional terrestrial MAGs, and a SAG obtained from groundwaters (Supplementary Fig. S9). Missing V-ATPase subunits in these *Nitrosomirales* genomes may reflect genome incompleteness.

The V-type ATPase has been proposed to serve to maintain the cytosolic pH homeostasis in acidophilic and acid tolerant AOA as well as deep marine AOA by pumping out excessive cytoplasmic protons under acidic or high-pressure conditions [95]. Recent AOA comparative population genomics and phylogenomic analyses have shown that V-type ATPase genes were widely distributed among deep-sea water column [14] and sedimentary [85] *Nitrosopumilales* AOA and desert *Nitrososphaerales* AOA populations [96]. In addition to the experimentally tested function in low pH adaptation, AOA V-type ATPase may be also coupled to sodium (Na^+^) motive force at high pH levels, protecting cells from high-salt stress [27, 96]. The ATPase subunit phylogenetic trees show that the AOA V-type ATPase subgroup is rooted with *Nitrosomirales* AOA variants (Supplementary Fig. S8). Thus, it is most likely that *Nitrosomirales* species also acquired the V-ATPase by horizontal gene transfer, as observed for other AOA order species [95]. The V-type *atp* operon was located next to the A-type *atp* operon in *Nitrosomirales* MAGs (Supplementary Fig. S9). In contrast, although the hadopelagic *Nitrosopumilales* AOA species also contain both types of ATPases, the V-type *atp* operon was distantly located from the A-type *atp* operon (Supplementary Fig. S9), suggesting the highly mobile V-type *atp* operon may have been reshuffled by several successive events along the evolutionary history of AOA.

Nitric oxide (NO) has been shown as a central intermediate in the archaeal ammonia oxidation pathway [7, 97], and the putative NO-forming nitrite reductase (NirK) is conserved among *Nitrosopumilales* and *Nitrososphaerales* AOA species [14]. However, no gene encoding NirK proteins has yet been identified in *Ca.* Nitrosocaldus AOA species [61, 62]. In contrast, it was identified in thermophilic *Ca.* Nitrosothermus AOA MAGs [83]. NirK homologs were also identified in both marine and terrestrial *Nitrosomirales* genomes, and many *Nitrosomirales* species encode two NirK paralogs in the genomes (Supplementary Fig. S10 and Supplementary Table S8). This could potentially signify evolutionary redundancy or specialization, thereby ensuring the consistent production of NO, an important intermediate in archaeal ammonia oxidation, through nitrite reduction across various environmental conditions. The *nirK* genes of the deep-sea *Nitrosomirales* MAG MTA5 and warm spring MAG WS3 did not cluster with those encoded by the relatively closely related *Ca*. Nitrosothermus AOA, but rather grouped with those of the distinctly related deep-sea water column B and terrestrial *Nitrosotenuis* AOA, respectively (Supplementary Fig. S10). This suggests that the *nirK* genes in these *Nitrosomirales* MAGs have been acquired via lateral gene transfer from *Nitrosopumilales* AOA that share similar habitats.

In addition, *ureC*, the gene encoding the alpha subunit of urease, was also found in terrestrial *Nitrosomirales* MAGs (Supplementary Fig. S11) and putatively acquired laterally from other mesophilic terrestrial AOA lineages. The *ureC* genes of *Nitrosomirales* MAGs were phylogenetically distinct from those of thermophilic *Ca.* Nitrosocaldales AOA but nested among *Nitrososphaerales* AOA lineages (Supplementary Fig. S11). Thus, *Nitrosomirales* AOA may have acquired urea utilization genes to enhance metabolic versatility during their evolution. Taken together, our results highlight the lateral transfer of several key genes and operons involved in energy conservation in *Nitrosomirales* AOA. Further culture-based and field studies are warranted to investigate whether the acquisition of these essential metabolic genes has facilitated their radiation into a diversity of subsurface, marine, and geothermal environments, as now revealed by our metagenome-based biogeography analysis.

### Each of the four AOA orders spans both marine and terrestrial habitats

Our comprehensive single-gene and whole-genome based phylogenetic analyses with additional *Nitrosomirales* genomes and marine AOA 16S rRNA sequences provide a better resolved framework of the AOA phylogeny and suggest that AOA have colonized moderate temperature environments multiple times over their evolutionary history. We found that the common ancestor of AOA diverged via two primary paths of evolution, ultimately giving rise to the two major contemporary branches. One of these branches constitutes the (hyper)thermophilic *Ca*. Nitrosocaldales AOA, along with the newly defined *Nitrosomirales* AOA, primarily found in terrestrial subsurface environments, abundant in some sponge-associated microbiomes, and occasionally detected in ocean waters. The second branch encompasses the previously described *Nitrososphaerales* that are mostly prevalent in terrestrial settings, and *Nitrosopumilales* that are mainly found in marine settings. (Fig. 1).

Previous molecular dating analyses suggested that AOA first transitioned into the temperate terrestrial environments before expanding to marine environments, and further transition from shallow marine into deep-sea waters awaited the oxygenation of the deep ocean during the Neoproterozoic [19, 47]. However, we found the presence of thermophilic *Nitrosocaldales* 16S rRNA gene sequences in various high-temperature marine environments, including shallow-sea hydrothermal vents [98], a coastal hot spring (NCBI accession No.: JX047158), deep-sea hydrothermal fields of the Mariana Trough [99], and the walls of an active deep-sea sulfide chimney with 302°C venting liquid [100] (Supplementary Fig. S12). As more marine *Nitrosocaldales* amplicon sequences, genomes, and cultures become available in the future, it will be interesting to investigate the distribution, relative abundance, adaptive features, and biogeochemical significance of *Nitrosocaldales* AOA in these (hyper)thermophilic marine environments. Further phylogenomic and molecular dating analyses of marine thermophilic *Nitrosocaldales* genomes will ultimately validate whether the expansion of AOA into both shallow and deep-sea habitats occurred prior to their transition to temperate terrestrial environments, which might push the origin of marine AOA further back in evolutionary history than previously hypothesized. In addition, we found *Nitrososphaerales* 16S rRNA sequences were present in many sponge and coral reef samples [76-78, 101] as well as deep-sea sediments [102] (Supplementary Fig. S12). This aligns with the comprehensive analysis of the distribution of *Nitrososphaerales amoA* genes [25], providing additional support for the expanded habitat range of *Nitrososphaerales* AOA, encompassing both terrestrial and marine environments. Taken together, these results indicate that each of the four AOA orders spans both marine and terrestrial environments (Supplementary Fig. S12). It is plausible that the terrestrial–marine habitat expansion occurred independently within each order, with some possibly reflecting late colonization events [18], depicting the dynamic and ongoing evolutionary process of this globally widespread functional guild.

## Conclusions

Our comparative genomic and phylogenomic analyses of 161 AOA and non-ammonia-oxidizing *Thaumarchaeota* genomes revealed a new AOA cluster, *Ca*. Nitrosomirales, that forms an order-level lineage within the class *Nitrososphaeria*. In addition to containing expected gene inventories for ammonia oxidation, carbon dioxide fixation, and B-vitamin biosynthesis, *Nitrosomirales* AOA have a genetic capacity consistent with the use of formate as a source of reductant and nitrate as an alternative electron acceptor, which may provide metabolic versatility under ammonia and oxygen deprivation. Biogeographic analyses of 16S rRNA and *amoA* genes as well as metagenomes together indicate that *Nitrosomirales* AOA are widely distributed in geothermal, terrestrial, and marine environments. They appear to represent the dominant type of AOA in a number of terrestrial subsurface environments and some deep-sea sponges. Evidence for the expansion of each of the four AOA orders into both terrestrial and marine habitats should foster a more detailed understanding of the early evolution and adaptive radiation of archaeal ammonia oxidation during the Proterozoic era.

## Supplementary Material

Supplementary_Dataset_S1_wrad002

Supplementary_Dataset_S2_wrad002

Supplementary_Dataset_S3_wrad002

Supplementary_Dataset_S4_wrad002

Table_S1_wrad002

Table_S2_wrad002

Table_S3_wrad002

Table_S4_wrad002

Table_S5_wrad002

Table_S6_wrad002

Table_S7_wrad002

Table_S8_wrad002

Table_S9_wrad002

SI_combined_wrad002
