# Supplementary material for: Novel order-level lineage of ammonia-oxidizing archaea widespread in marine and terrestrial environments
Source: ISME J. 2024 Jan 10;18(1):wrad002. doi: 10.1093/ismejo/wrad002 (PMC10811736; doi:10.1093/ismejo/wrad002)
Supplement: SI_combined_wrad002 [file si_combined_wrad002.pdf]

Figure S1

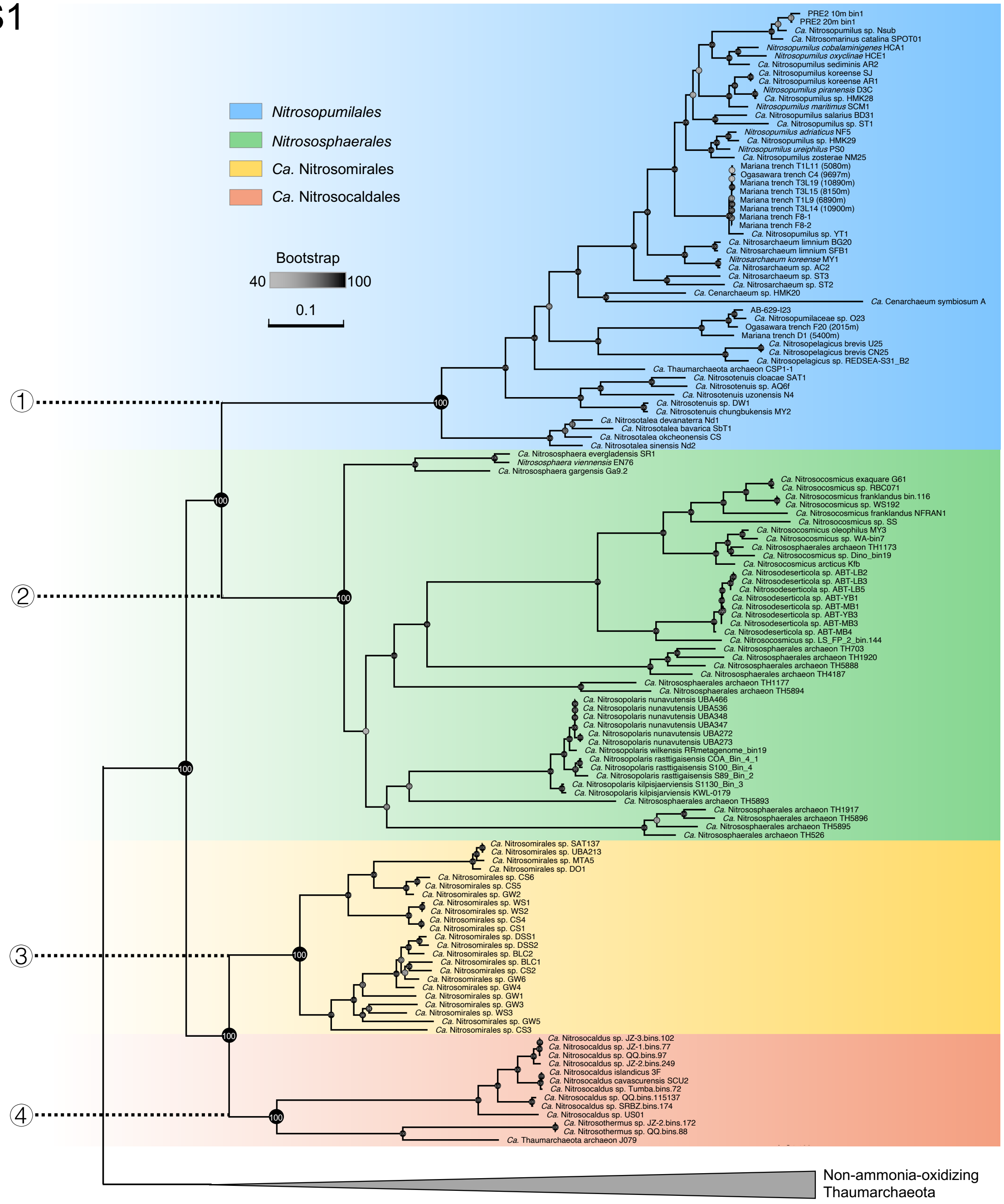

Figure S1. Phylogenomic inference of *Ca*. Nitrosomirales and other AOA orders. The color scheme of each AOA order and non-ammonia-oxidizing Thaumarchaeota outgroup were the same as those depicted in Figure 1. Phylogenomic tree was constructed using the best-fit model (LG+F+R9, single amino acid replacement rate matrix model). Confidence values were provided based on 1,000 bootstrap replications.

Figure S2

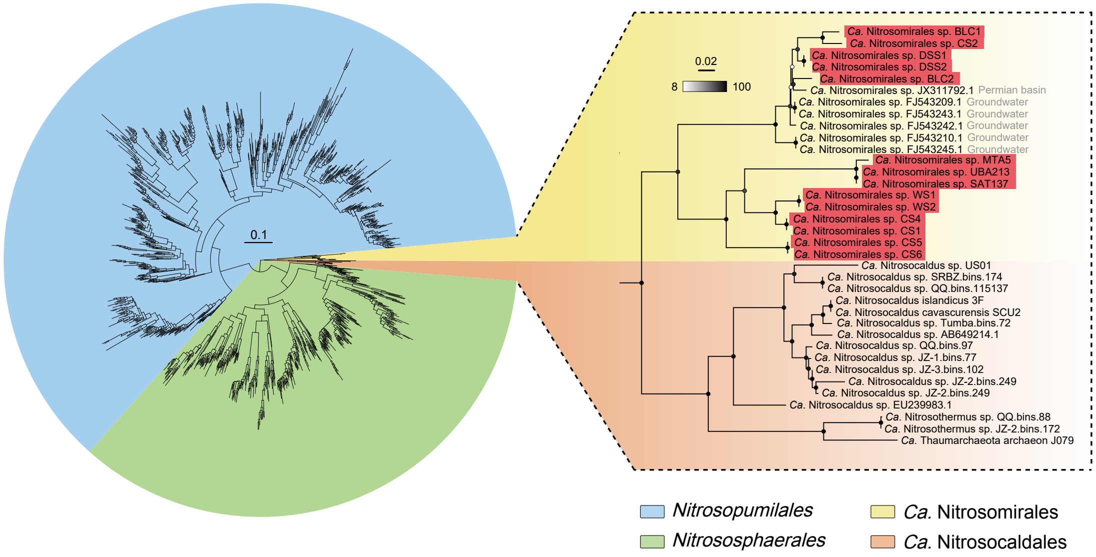

Figure S2. Phylogenetic inference of AOA *amoA* genes. A zoomed-in section was presented to show the detailed phylogeny of the basal sister lineages *Ca*. Nitrosomirales and *Ca*. Nitrosocaldales. The *amoA* genes that were retrieved from *Ca*. Nitrosomirales genomes were highlighted in red. The scale bars represent 10% and 2% estimated sequence divergence for the overall AOA tree and zoomed-in tree, respectively. Confidence values were provided based on 1000 bootstrap replications.

Figure S3

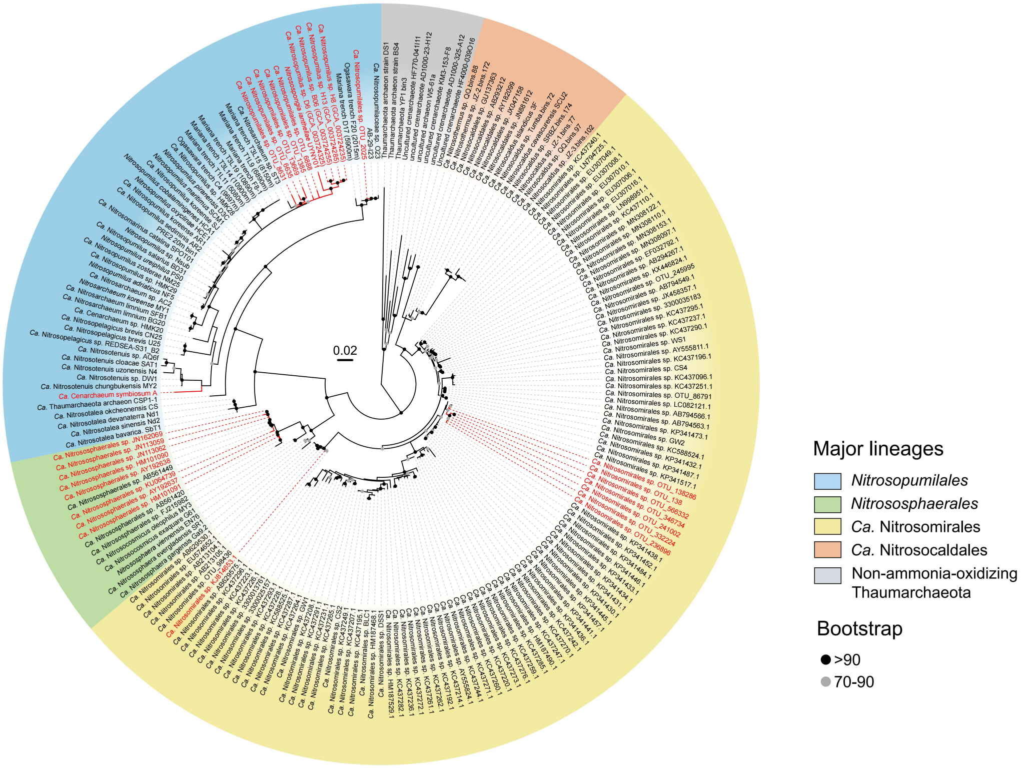

Figure S3. The distribution of marine sponge-associated AOA 16S rRNA genes among the *Nitrosopumilales*, *Nitrososphaerales*, and *Ca*. Nitrosomirales. These genes were highlighted in red on the 16S rRNA gene tree.

Figure S4

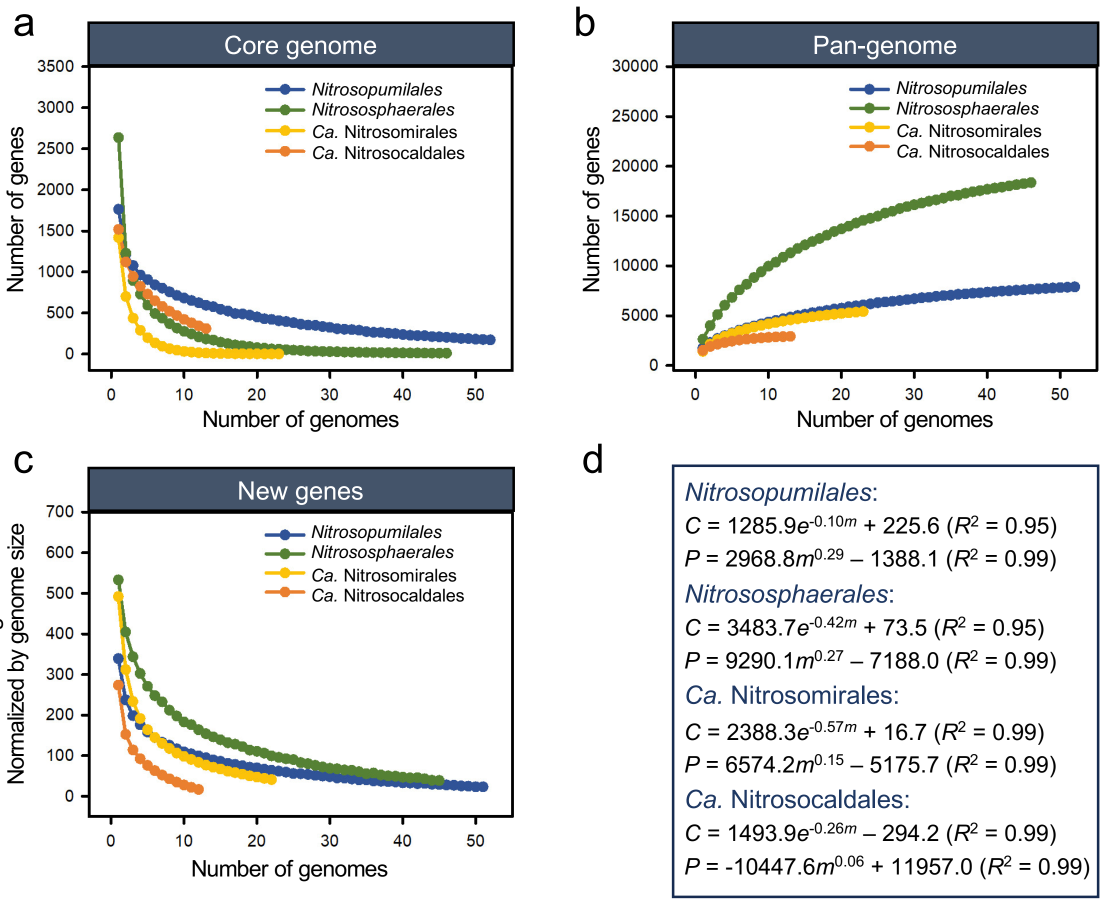

Figure S4. The sizes of core (**a**) and pan-genome (**b**) as well as the number of new unique genes (**c**) of four AOA orders. Fifty-two *Nitrosopumilales* genomes, 46 *Nitrososphaerales* genomes, 23 *Ca*. Nitrosomirales genomes, and 13 *Ca*. Nitrosocaldales genomes were used for calculation. For *m* genomes selected out of *n* strains, a total of *n!/ m!·(n-m)!* combinations were calculated to determine the core and pan-genomes as well as new gene numbers. The average number of new unique genes was normalized by the size of selected genomes (Mb). The normalized value from each combination was profiled as the number of *m* genomes sequentially added. Each data point represents the average value of all combinations. The core and pan-genomes were fitted using Heaps' law (**d**), where *C* denotes the number of genes in core genome, *P* denotes the number of genes in pan-genome, and *m* denotes the number of genomes.

Figure S5

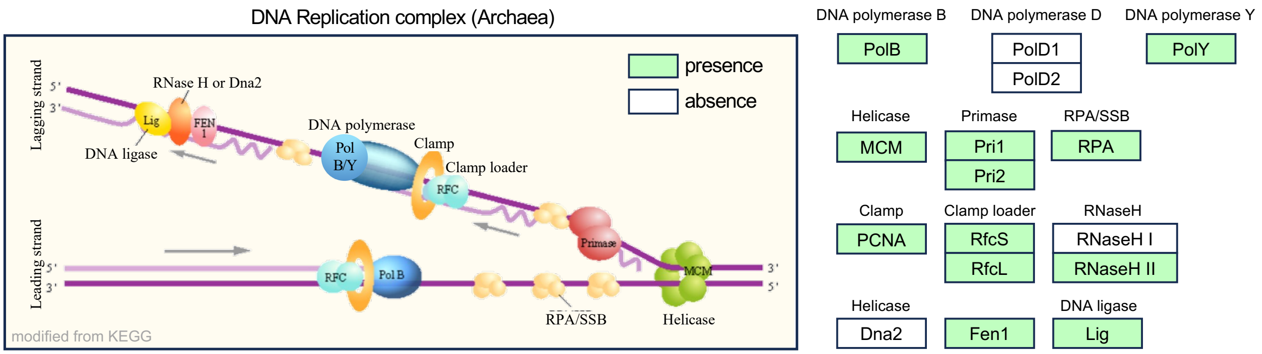

Figure S5. The schematic diagram illustrating the DNA replication components of *Ca*. Nitrosomirales AOA.

Figure S6

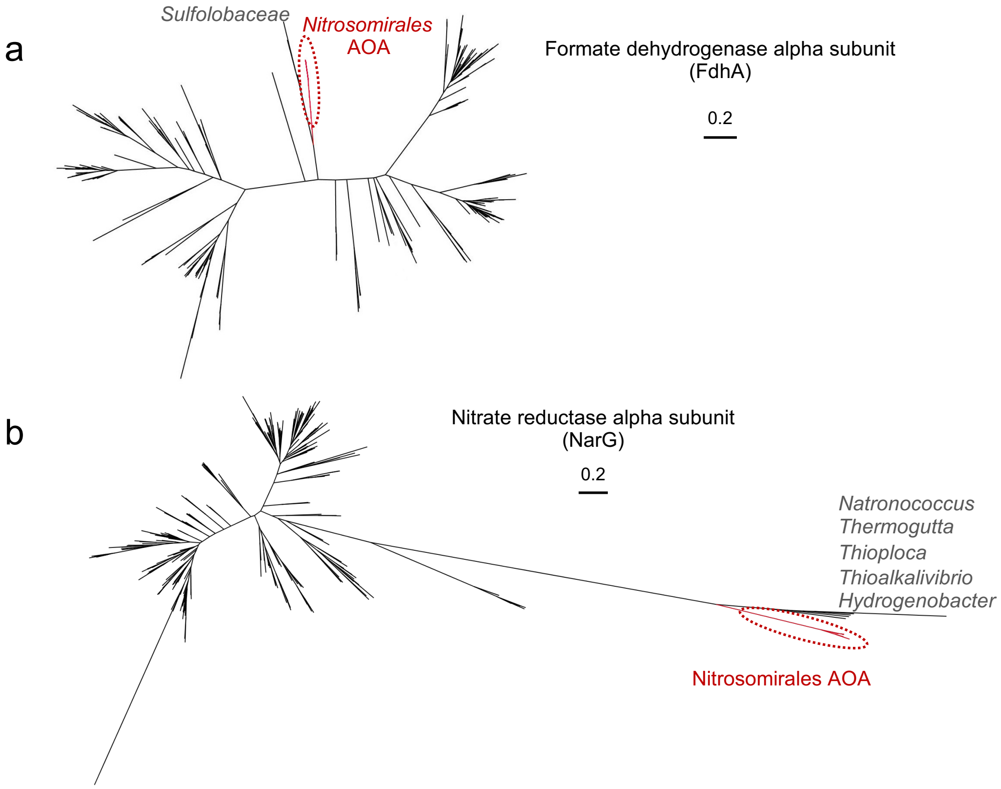

Figure S6. Phylogenetic trees of formate dehydrogenase alpha subunit FdhA **(a)** and nitrate reductase alpha subunit NarG **(b)**. The FdhA and NarG protein clusters of *Ca*. Nitrosomirales AOA, consisting of 6 sequences and 8 sequences, respectively (highlighted in red in the phylogenetic trees), were aligned with the reference sequences from UniProt database using MAFFT (version 7.407). The alignment was trimmed with Gblocks (version 0.91b), and a maximum likelihood phylogenetic tree was constructed using IQTREE (version 2.1.2 COVID-edition) with 1,000 ultrafast bootstrap replicates. The scale bar represents the number of amino acid substitutions per site.

Figure S7

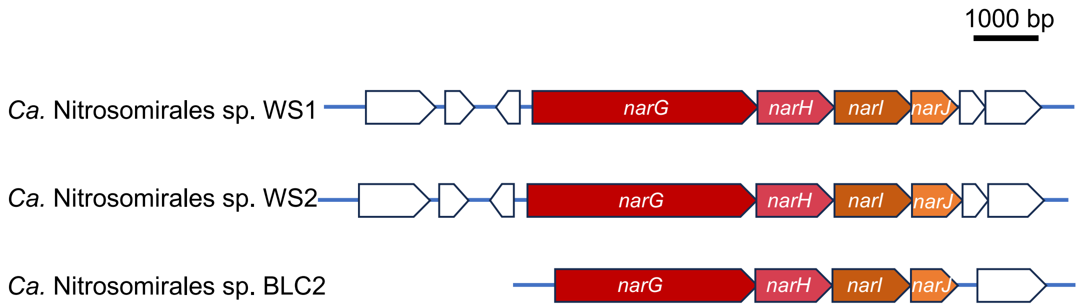

Figure S7. Schematic illustration of *nar* operon representatives of *Ca*. Nitrosomirales AOA. Homologous genes are shown by the same color.

Figure S8

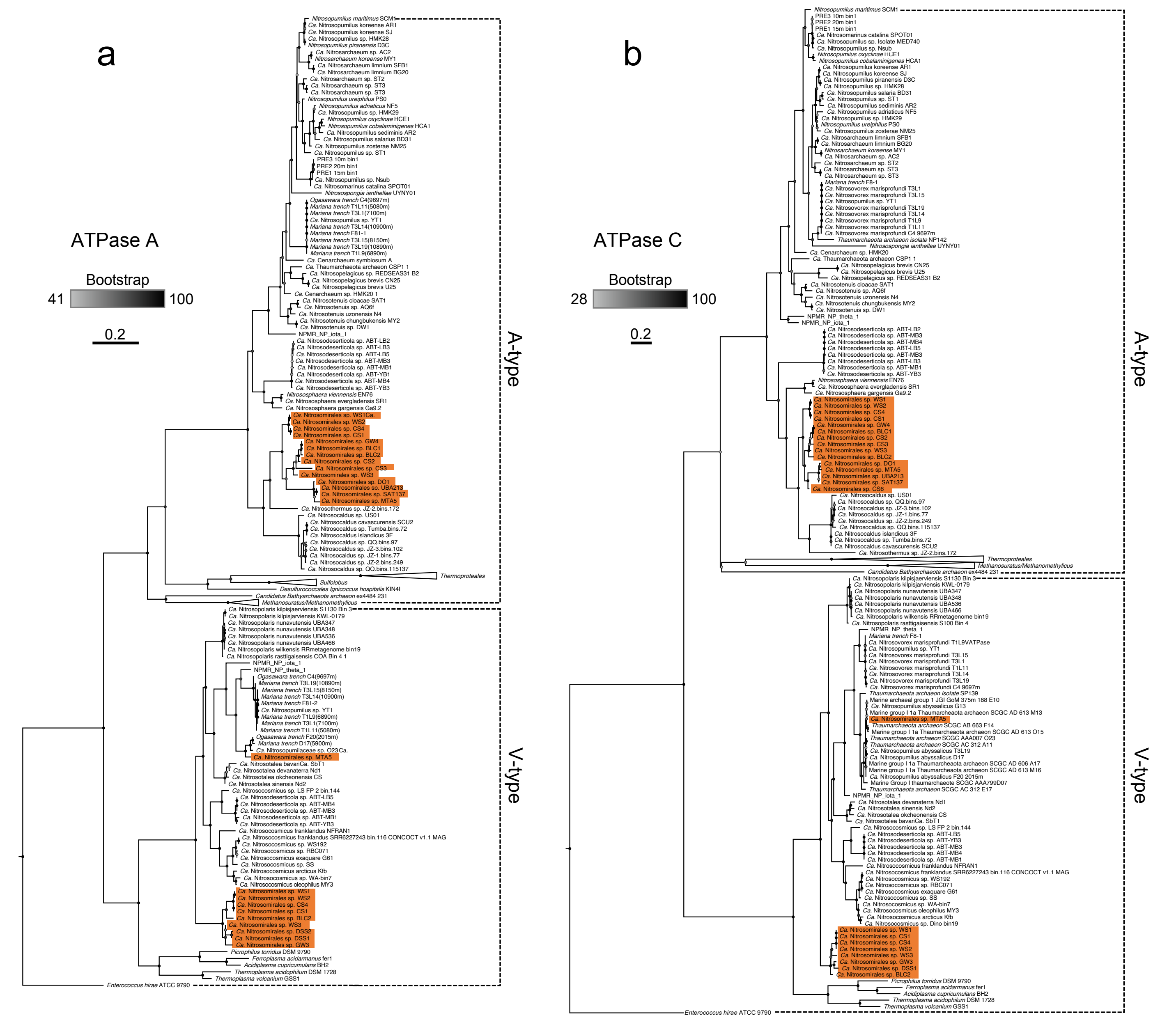

Figure S8. Phylogeny of AOA *atpA* (a) and *atpC* (b) encoding the alpha and epsilon units of ATPase, respectively. The orange shading highlights the *atpA* and *atpC* genes of *Ca*. Nitrosomirales AOA. Confidence values are based on 1000 bootstrap replications. The scale bars represent 0.2 substitutions per nucleotide position.

Figure S9

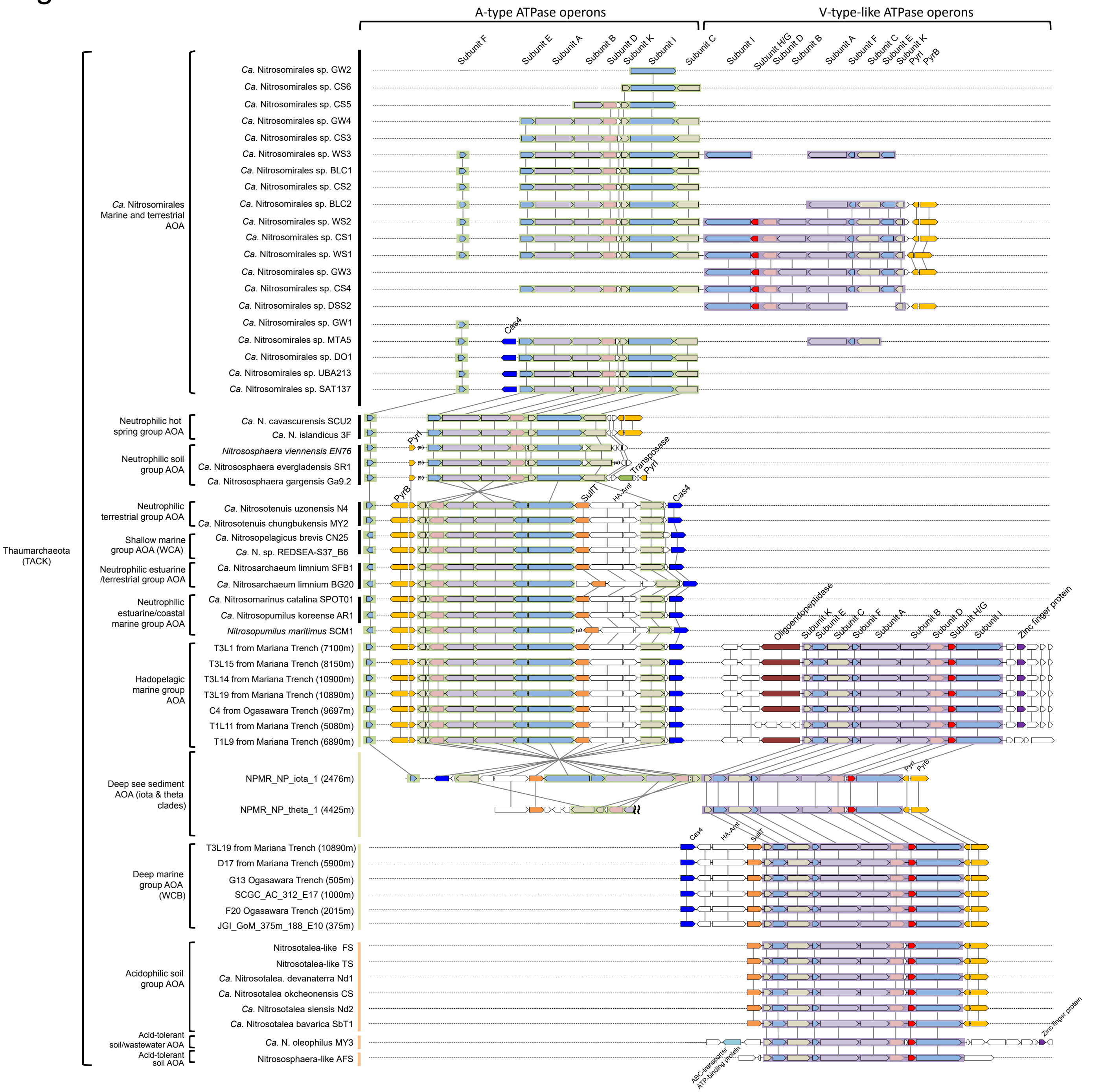

Figure S9. Schematic depiction of *atp* operons in *Ca*. Nitrosomirales AOA and other AOA lineages. Homologous genes are colorcoded and linked by connecting lines. Transcriptional orientation is indicated by arow directions The designations WCA and WCB correspond to water column A and water column B marine AOA, respectively. Specific protein abbreviations include PyrB (aspartate carbamoyltransferase), PyrI (regulatory subunit of aspartate carbamoyltransferase), SulfT (thiosulfate/3mercaptopyruvate sulfurtransferase), HA-Amt (high-affinity ammonia transporter) and Cas4 (CRISPR-associated exonuclease Cas4). The numeral enclosed in braces denotes the count of unrepresented genes.

Figure S10

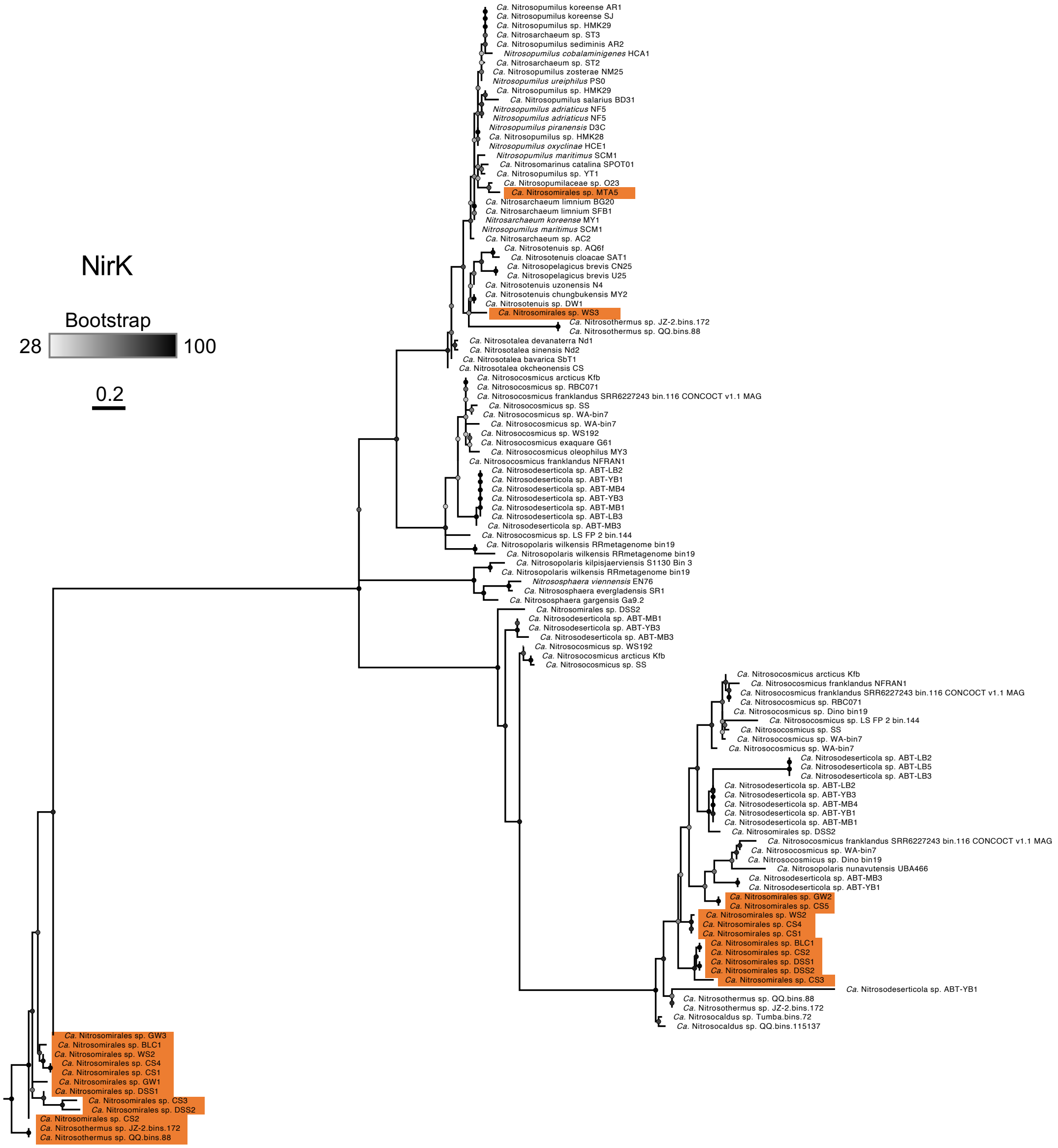

Figure S10. Phylogeny of AOA *nirK* (encoding the putative copper-dependent nitrite reductase). The orange shading highlights the *Ca*. Nitrosomirales *nirK* genes. Confidence values are on the basis of 1000 bootstrap replications. The scale bar represents 0.2 substitutions per nucleotide position.

Figure S11

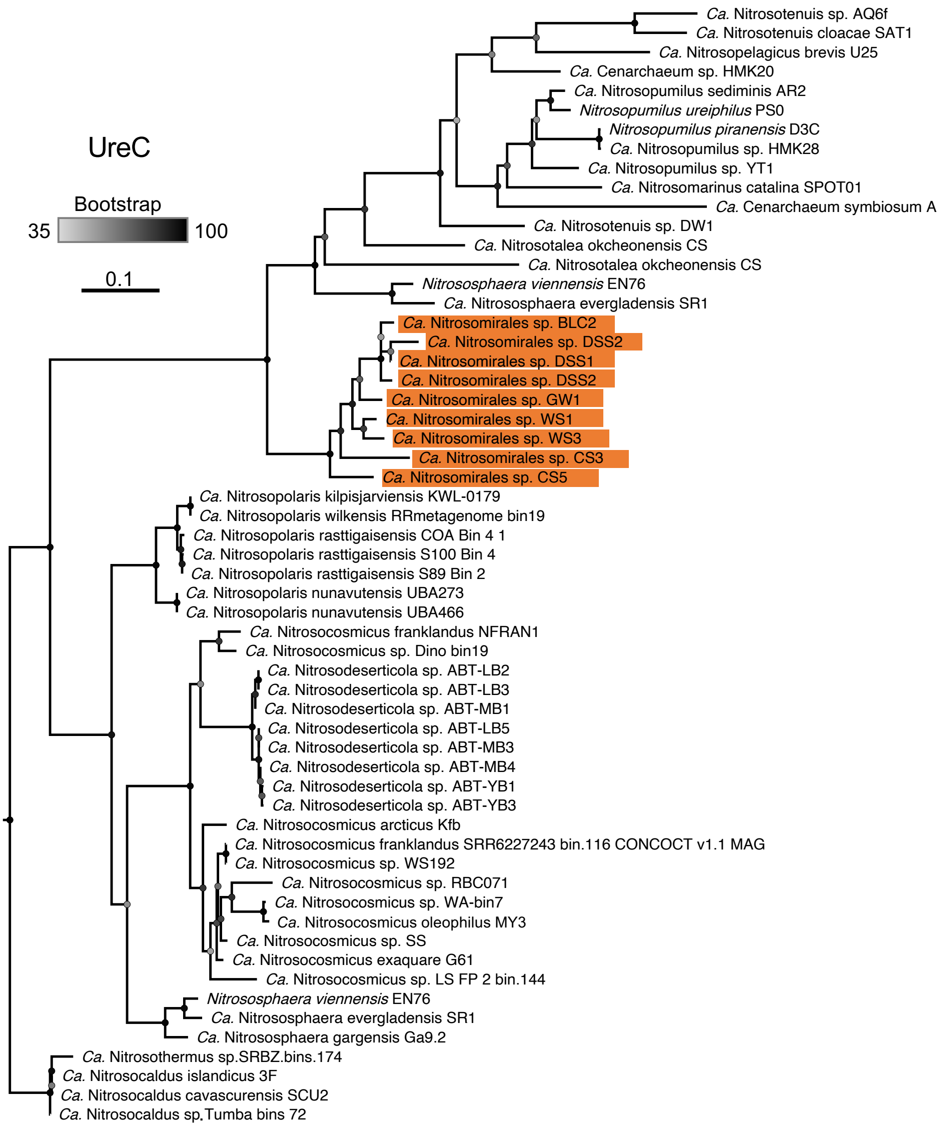

Figure S11. Phylogeny of AOA *ureC* (encoding the alpha subunit of urease). The orange shading highlights the *ureC* genes of *Ca*. Nitrosomirales AOA. Confidence values are based on 1000 bootstrap replications. The scale bar represents 0.1 nucleotides changes per position.

Figure S12

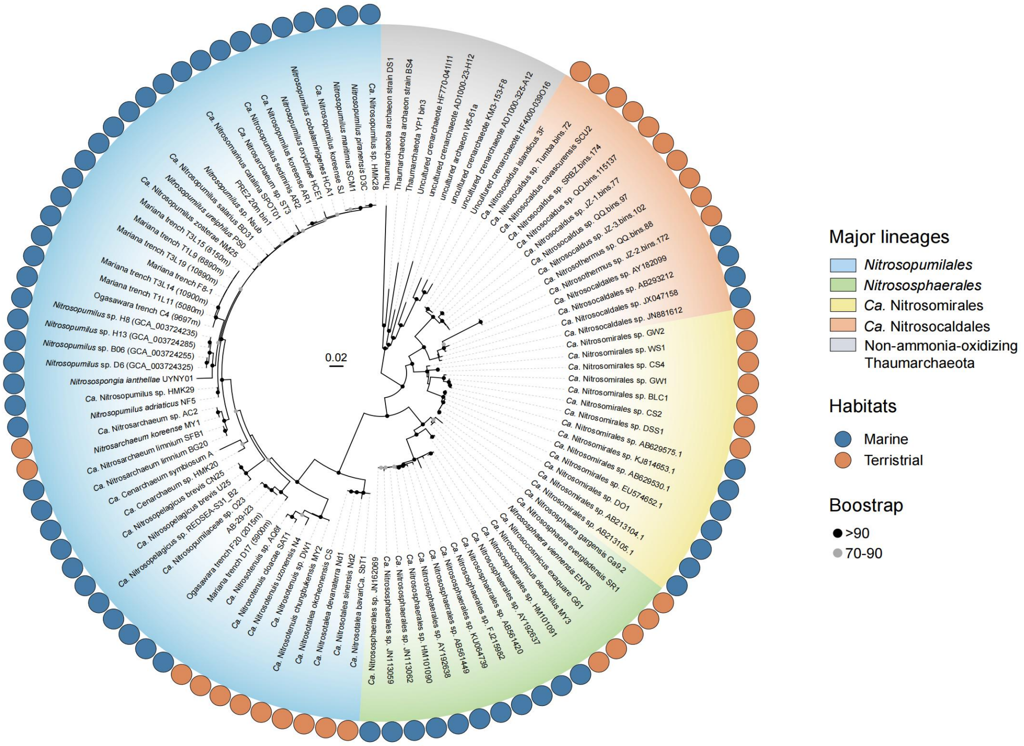

Figure S12. Both marine (blue circles) and terrestrial (brown circles) AOA 16S rRNA genes were found within each AOA order. The scale bar represents 2% estimated sequence divergence.

Table S1. The list of ammonia-oxidizing and non-ammonia-oxidizing *Thaumarchaeota* genomes used in this study. Genome sequence data are available through accession numbers in NCBI, JGI, BIGD, or DDBJ databases.

Table S2. The 70 genes shared among AOA genomes and outgroup archaeal genomes that were used to construct the concatenated phylogenomic tree. NCBI accession numbers for *Nitrosopumilus maritimus*, *Cenarchaeum symbiosum*, and *Caldarchaeum subterraneum* were given to represent each conserved gene.

Table S3. Summary genomic information of *Nitrosomirales* MAGs and SAGs.

Table S4. Average nucleotide identity (ANI) and the fraction of genomes shared between each pairwise comparison of AOA genomes used in this study.

Table S5. Average amino acid identity (AAI) between each pairwise comparison of AOA genomes used in this study.

Table S6. Relative abundance of *Ca*. Nitrosomirales AOA and other groups of AOA in the 16S rRNA gene amplicon sequencing samples collected from various terrestrial and marine environments.

Table S7. Relative abundance of *Ca*. Nitrosomirales AOA and other groups of AOA in the metagenome samples collected from various terrestrial and marine environments.

Table S8. Distribution of major metabolic pathway genes in *Nitrosomirales* AOA genomes. The gene sets were organized and compiled according to the previous comparative genomics studies of marine [1, 2, 3, 4], soil [5], and hot spring [6, 7] AOA species.

Table S9. Matrix and arCOGs annotation of the enriched orthologous groups in *Nitrosomirales* AOA (protein clusters A and B) as shown in Figure 4.

Supplementary Dataset S1. In-house AOA *amoA* gene database.

Supplementary Dataset S2. The GFF file of *Ca.* Nitrosomirales sp. UBA213.

Supplementary Dataset S3. The GFF file of *Ca.* Nitrosomirales sp. WS3.

Supplementary Dataset S4. The contig sequences of *Ca.* Nitrosomirales sp. WS3.

References

1. Qin W, Zheng Y, Zhao F, Wang YL, Urakawa H, Martens-Habbena W *et al* (2020). Alternative strategies of nutrient acquisition and energy conservation map to the biogeography of marine ammonia-oxidizing archaea. ISME J **14:** 2595-2609.

2. Qin W, Amin SA, Lundeen RA, Heal KR, Martens-Habbena W, Turkarslan S *et al* (2018). Stress response of a marine ammonia-oxidizing archaeon informs physiological status of environmental populations. ISME J **12:** 508-519.

3. Santoro AE, Dupont CL, Richter RA, Craig MT, Carini P, McIlvin MR *et al* (2015). Genomic and proteomic characterization of *Candidatus* Nitrosopelagicus brevis: An ammonia-oxidizing archaeon from the open ocean. Proc Natl Acad Sci USA **112:** 1173-1178.

4. Walker CB, de la Torre JR, Klotz MG, Urakawa H, Pinel N, Arp DJ *et al* (2010). *Nitrosopumilus maritimus* genome reveals unique mechanisms for nitrification and autotrophy in globally distributed marine crenarchaea. Proc Natl Acad Sci USA **107:** 8818-8823.

5. Kerou M, Ponce-Toledo RI, Zhao R, Abby SS, Hirai M, Nomaki H *et al* (2021). Genomes of Thaumarchaeota from deep sea sediments reveal specific adaptations of three independently evolved lineages. ISME J **15:** 2792-2808.

6. Abby SS, Melcher M, Kerou M, Krupovic M, Stieglmeier M, Rossel C *et al* (2018). *Candidatus* Nitrosocaldus cavascurensis, an ammonia oxidizing, extremely thermophilic archaeon with a highly mobile genome. Front Microbiol **9:** 28.

7. Daebeler A, Herbold CW, Vierheilig J, Sedlacek CJ, Pjevac P, Albertsen M *et al* (2018). Cultivation and genomic analysis of "*Candidatus* Nitrosocaldus islandicus," an obligately thermophilic, ammonia-oxidizing Thaumarchaeon from a hot spring biofilm in Graendalur Valley, Iceland. Front Microbiol **9:** 193.
